# Germline *PTPRD* Mutations in Ewing Sarcoma: Biologic and Clinical Implications

**DOI:** 10.18632/oncotarget.1021

**Published:** 2013-06-05

**Authors:** Yunyun Jiang, Filip Janku, Vivek Subbiah, Laura S. Angelo, Aung Naing, Peter M. Anderson, Cynthia E. Herzog, Siqing Fu, Robert S. Benjamin, Razelle Kurzrock

**Affiliations:** ^1^ Department of Investigational Cancer Therapeutics (Phase I Clinical Trials Program), The University of Texas MD Anderson Cancer Center, Houston, Texas, USA; ^2^ Department of Pediatrics, The University of Texas MD Anderson Cancer Center, Houston, Texas, USA; ^3^ Department of Sarcoma Oncology, The University of Texas MD Anderson Cancer Center, Houston, Texas, USA; ^4^ Moores Cancer Center, The University of California San Diego, La Jolla, California, USA

**Keywords:** Ewing sarcoma, PTPRD, mutation, germline

## Abstract

Ewing sarcoma occurs in children, adolescents and young adults. High STAT3 levels have been reported in approximately 50% of patients with Ewing sarcoma, and may be important in tumorigenesis. *Protein tyrosine phosphatase delta* (*PTPRD*) is a tumor suppressor that inhibits STAT3 activation. To date, while somatic mutations in *PTPRD* have been reported in diverse tumors, germline mutations of *PTPRD* have not been investigated in Ewing sarcoma or other cancers. We identified a novel germline mutation in the *PTPRD* gene in three of eight patients (37.5%) with metastatic Ewing sarcoma. Although the functional impact in two of the patients is unclear, in one of them the aberration was annotated as a W775stop germline mutation, and would be expected to lead to gene truncation and, hence, loss of the STAT3 dephosphorylation function of *PTPRD*. Since STAT3 is phosphorylated after being recruited to the insulin growth factor receptor (IGF-1R), suppression of IGF-1R could attenuate the enhanced STAT3 activation expected in the presence of *PTPRD* mutations. Of interest, two of three patients with germline PTPRD mutations achieved durable complete responses following treatment with IGF-1R monoclonal antibody-based therapies. Our pilot data suggest that *PTPRD* germline mutations may play a role in the development of Ewing sarcoma, a disease of young people, and their presence may have implications for therapy.

## INTRODUCTION

Germline mutations occur in several cancer-related genes including, but not limited to, *BRCA1*, *BRCA2*, *RB1* and *TP53*.[1-4] Patients with these mutations often develop tumors at a young age. Ewing sarcoma is a malignant, small, round blue-cell tumor that primarily occurs in children, adolescents and young adults. [5, 6] It is associated with a somatic translocation of chromosome 22, which typically generates a fusion protein product designated as EWS-FLI1.[7]

Approximately 50% of patients with Ewing sarcoma also express activated/phosphorylated signal transducer and activator of transcription 3 (STAT3).[8] STAT3 is known to drive pathways regulating normal cell growth, which result in tumorigenesis when dysregulated.[9] Protein tyrosine phosphatase delta (PTPRD), a tumor suppressor belonging to the protein tyrosine phosphatase (PTP) family[10], plays an essential role in dephosphorylating STAT3.[11] Genetic aberrations of *PTPRD* are associated with a poor prognosis in malignant tumors.[11, 12] Mutations in *PTPRD* have been reported in approximately 13% of head and neck squamous cell carcinoma [11], 12% of melanoma [13], and in a small subset of various other malignancies.[11] However, germline mutations of *PTPRD* have not been previously described.

Here we report three of eight patients (37.5%) with metastatic Ewing sarcoma who harbored germline mutations in the *PTPRD* gene. Using next-generation sequencing (NGS), two germline mutations were found in one patient, including one leading to truncation and subsequent loss of function of the PTPRD suppressor. Of interest, two of these three patients achieved a complete response (CR) following insulin-like growth factor 1 receptor (IGF-1R) inhibitor-based therapy. Because phosphorylated STAT3 is frequently upregulated in Ewing sarcoma [8] and PTPRD dephosphorylates STAT3, the role of germline and somatic *PTPRD* mutations in Ewing sarcoma as well as the implications for IGF-1R targeted therapy warrant exploration.

## RESULTS

A total of eight patients diagnosed with advanced/metastatic Ewing sarcoma with available PBMCs who had been referred to the CCTT and/or Department of Pediatrics at MD Anderson were analyzed. The median age at diagnosis was 19.5 years (range, 13 to 34 years). All patients developed metastatic disease between 0 and 7 years after initial diagnosis (Table 1).

**Table 1:** Patients with Ewing sarcoma tested for germline mutations in PTPRD and outcomes with IGF-1R-based therapy

	Age at diagnosis	Mutation	Time from diagnosis metastasis	Best RECIST response to IGF-1R inhibitor monotherapy (PFS)	Best RECIST response to IGF-1R+mTOR inhibitor (PFS)
1	24	V253I, W775stop	6 months	CR, −100% (3 years)	CR, −100% (2 years)
2	22	T781A	0	Not treated	PD, +21%
3	33	Wild-type	7 years	Not treated	SD, −27% (16+ months)
4	18	Wild-type	2 years	Not treated	SD, −23% (20 months)
5	13	R995C	1 years	Not treated	CR, −100% (28 months)
6	21	Wild-type	0	Not treated	PD, −42%, new lesion
7	13	Wild-type	0	Not treated	SD, −14% (4.5 months)
8	13	Wild-type	0	Not treated	Not treated

Abbreviations: RECIST, response evaluation criteria in solid tumors; IGF-1R, insulin-like growth factor receptor 1; CR, complete response; PD, progressive disease; SD, stable disease

**Figure 1:**
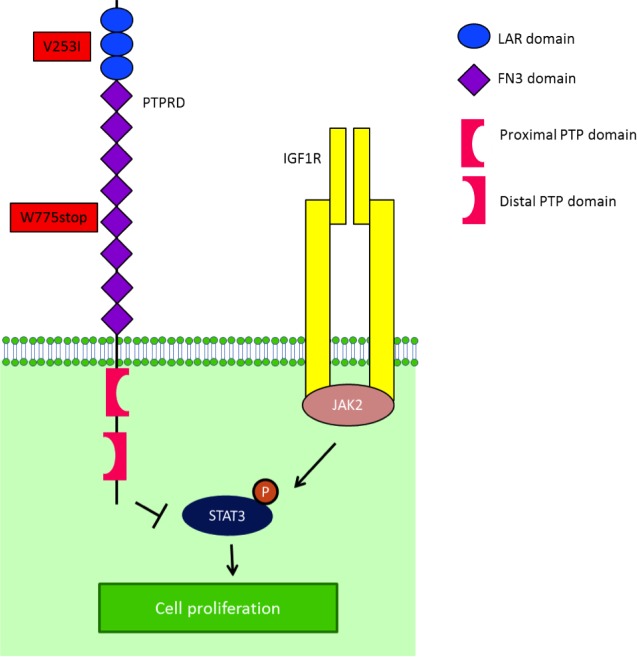
IGF-1R is one of the mediators of STAT3 activity STAT3 is phosphorylated by JAK2, after being recruited to IGF-1R by RACK1. After STAT3's phosphorylation by JAK2, PTPRD normally dephosphorylates STAT3. In the presence of a truncated PTPRD, STAT3 would remain phosphorylated.

**Figure 2:**
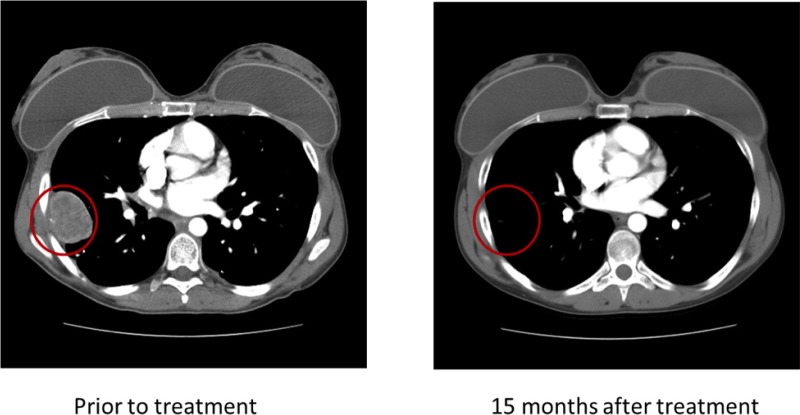
Patient 1 demonstrated a durable complete response to therapy with an IGF-1R inhibitor

Of the eight patients with Ewing sarcoma, three (37.5%) had germline mutations in the *PTPRD* gene. Patient 1 (Table 1; age 24 at diagnosis) had mutational analysis of 182 cancer-related genomic alterations in formalin-fixed paraffin-embedded tumor tissue performed using a Clinical Laboratory Improvement Amendment approved Foundation One platform. Simultaneously, DNA extracted from tumor tissue and PBMCs from the same patient were analyzed independently with next-generation whole exome sequencing in the MD Anderson Core Laboratory using the SOLiD platform. A *PTPRD* mutation annotated as a W775stop germline mutation was found in both the patient's tumor and PBMCs (Figure 1). The *PTPRD* mutation was confirmed by polymerase chain reaction (PCR)-based Sanger sequencing in genomic DNA derived from tumor and PBMCs. The W775stop germline mutation is located in the extracellular fibronectin type III (FN3) region (Figure 1).[10] The mutation of tryptophan to a stop codon results in the truncation of all transmembrane and intracellular domains, which leads to partial loss of the dephosphorylation function of PTPRD. Because PTPRD functions as a STAT3 phosphorylation suppressor, it is plausible that partial loss of PTPRD can lead to increased STAT3 phosphorylation.[11] In addition, NGS revealed a V253I germline mutation located in the third immunoglobulin (Ig)-like domain of the receptor protein tyrosine phosphatase (RPTP)-F region, also known as LAR, within the extracellular domain (Figure 1) [10]. The impact of this mutation is, however, unclear. Patient 1 had durable CRs resulting from IGF-1R inhibitor-based therapies (Figure 2).[14, 17-20]

PBMCs from an additional seven patients (2 to 8, Table 1) with advanced Ewing sarcoma were analyzed for *PTPRD* germline mutations in all 35 exons (exons 11-45, ENST00000381196) using Sanger sequencing, as previously described.[11] Among these seven patients, five had wild-type *PTPRD* and two had *PTPRD* germline mutations: T781A and R995C. The tumor tissues from these two patients were not available for testing. A second patient with a *PTPRD* mutation (Patient 5, Table 1) also attained a CR on IGF-1R inhibitor-based therapy.

## DISCUSSION

We identified germline *PTPRD* mutations in three (37.5%) of eight patients with advanced/metastatic Ewing sarcoma. The first patient had two mutations (Table 1). The *PTPRD* W775stop germline mutation is expected to express truncated PTPRD protein, leading to loss of function; however, it has not previously been reported in the Exome Sequencing Project (ESP) [21] and Catalogue of Somatic Mutations in Cancer (COSMIC) databases [22] in the general population or in cancer patients (Figure 1). The W775stop germline mutation can lead to the truncation of all transmembrane and intracellular domains, which can putatively result in partial loss of the dephosphorylation function of PTPRD. Because PTPRD serves to suppress STAT3 phosphorylation [11], partial loss of PTPRD can possibly lead to increased STAT3 phosphorylation, which has been reported in approximately 50% of patients with Ewing sarcoma.[8] Patient 1 also had a PTPRD V253I germline mutation located within the extracellular domain coding region; its functional consequences are unclear (Figure 1).[10] In another two patients, we identified germline mutations T781A and R995C, which are single nucleotide polymorphisms of the PTPRD suppressor that have been reported in a small subset (2.2% and 7.4%, respectively) of the general population [21], but whose functional impact is unclear.

Two of three patients with germline *PTPRD* mutations attained a CR in response to therapies with a IGF-1R monoclonal antibody and or a combination of an IGF-1R monoclonal antibody and the mTOR inhibitor temsirolimus. IGF-1R is one of the mediators of STAT3 activity. STAT3 is phosphorylated by JAK2, after being recruited to the IGF-1R by RACK1 (Figure 1).[23] After STAT3 phosphorylation by JAK2, PTPRD normally serves to dephosphorylate STAT3. In the presence of truncated PTPRD, STAT3 would remain phosphorylated. However, based on the biology described, an IGF-1R inhibitor might interfere with the initial phosphorylation of STAT3, perhaps explaining the response to IGF-1R inhibition in these patients.

Somatic *PTPRD* mutations have been identified in a small subset of patients with cancer types such as glioblastoma multiforme, melanoma, squamous cell carcinoma of the head and neck, and others.[11, 13] To date, germline mutations of *PTPRD* have not been described in Ewing sarcoma. Our sample size is small, and the functional consequences of the mutations have not been fully elucidated; therefore, our findings should be interpreted with caution. However, the role of germline *PTPRD* mutations in predisposition to Ewing sarcoma, a disease of children, adolescents, and young adults, and the implications of these mutations for therapy with IGF-1R and STAT3 inhibitors warrants further investigation.

## PATIENTS AND METHODS

### Patients

We reviewed the electronic medical records of eight patients with advanced/metastatic Ewing sarcoma referred to the Clinical Center for Targeted Therapy (CCTT) at The University of Texas MD Anderson Cancer Center (MD Anderson) who had available peripheral blood mononuclear cells (PBMCs) and, in some cases, tumor tissue. Data were collected from transcribed notes, radiology films and reports in the electronic medical record and other source documentation. Registering patients in the database, clinical, pathologic, laboratory and pathology assessment were performed at MD Anderson. The study and all treatments were conducted in accordance with the guidelines of the MD Anderson Institutional Review Board.

### Molecular analysis

Targeted NGS of tumor tissue was performed in a Clinical Laboratory Improvement Amendment-approved laboratory at Foundation Medicine (Cambridge, MA). Genomic libraries were captured for 3,230 exons in 182 cancer-related genes plus 37 introns from 14 genes that are often rearranged in cancer and sequenced to an average median depth of 734X with 99% of bases covered >100X.

Whole exome sequencing of tumor and PBMC samples was performed using an Applied Biosystems 5500XL SOLiD Next-Generation Sequencing platform (Life Technologies, Carlsbad, CA) in the MD Anderson Core laboratory. The 5500XL SOLiD system generates 300 GB of mappable sequence tags in a single run. This level of data throughput enables 30X coverage of the human genome with high accuracy and depth, facilitating detection of rare somatic mutations, insertions and deletions in patient samples.

Sanger sequencing of tumor tissue and PBMCs used to identify *PTPRD* mutations in all 35 exon (exons 11-45, ENST00000381196) was done as previously described.[11]

### Treatment

Patients were enrolled in clinical trials with a single-agent IGF-1R monoclonal antibody or a combination of a monoclonal IGF-1R monoclonal antibody and the mTOR inhibitor temsirolimus.[14, 15] Treatment was carried out according to the specific requisites in these treatment protocols and continued until disease progression or unacceptable toxicity occurred.

Assessments, including history, physical examination, and laboratory evaluations, were performed as specified in each protocol, typically before the initiation of therapy, weekly during the first cycle, and then, at a minimum, at the beginning of each new treatment cycle. Response was assessed from computed tomography (CT) scans and/or magnetic resonance imaging (MRI) at baseline before treatment initiation and then every 2 cycles (8 weeks). All radiographs were read in the Department of Radiology at MD Anderson and reviewed in the Department of Investigational Cancer Therapeutics tumor measurement clinic. Responses were categorized per RECIST criteria.[16] In brief, a CR was defined as the disappearance of all measurable and non-measurable disease; partial response (PR) was defined as at least a 30% decrease in the sum of the longest diameter of measurable target lesions; progressive disease (PD) was defined as at least a 20% increase in the sum of the longest diameter of measurable target lesions, or unequivocal progression of a non-target lesion, or the appearance of a new lesion; and stable disease (SD) was defined as neither sufficient shrinkage to qualify as a PR nor an increase sufficient to qualify as PD.

### Statistical analysis

There was no formal statistical analysis; however, progression-free survival (PFS) was defined as the time interval from the start of therapy to the first observation of disease progression or death, whichever occurred first.
